# Assessing the resilience of the belt and road countries and its spatial heterogeneity: A comprehensive approach

**DOI:** 10.1371/journal.pone.0238475

**Published:** 2020-09-02

**Authors:** Shuai Zhang, Fan Zhang, Chengxin Wang, Zhaohan Wang

**Affiliations:** 1 College of Geography and Environment, Shandong Normal University, Jinan, Shandong, China; 2 Key Laboratory of Land Surface Pattern and Simulation, Institute of Geographic Sciences and Natural Resources Research, Chinese Academy of Sciences, Beijing, China; 3 Collaborative Innovation Center of Human-Nature and Green Development in Universities of Shandong, Jinan, Shandong, China; Northeastern University (Shenyang China), CHINA

## Abstract

Regional resilience refers to the resilience of a country or region against the ecological environment, social economy, and other internal and external natural factors and human factors in the process of development. When this resilience is lower than a certain critical threshold, the country or region will be in a fragile state. The comprehensive embodiment of ecological resilience, social resilience, and economic resilience of a country or region is regional resilience. Due to the wide range of countries along “the Belt and Road”, differences in natural background conditions and stages of economic and social development among different countries lead to different degrees of vulnerability, and the improvement of resilience is conducive to reducing vulnerability. At the same time, the research on the measurement and differentiation characteristics of regional resilience is of considerable significance to solve the weak foundation of environmental management and the lack of ability to deal with climate change of “the Belt and Road” countries. In this study, by using entropy weighting method and multi-index comprehensive evaluation method, 24 specific indicators are selected from three different dimensions: ecology, economy, and society, to construct a comprehensive evaluation index system about “the Belt and Road” countries resilience, and to evaluate the comprehensive resilience and spatial heterogeneity characteristics of China and 64 countries along “the Belt and Road”, and use multiple linear regression analysis to identify the main influencing factors of comprehensive resilience and analyze its influencing mechanism. According to the research, the overall resilience level of “the Belt and Road” countries shows prominent differentiation characteristics of “extreme difference”, the countries with low and low recovery status account for the vast majority; and the spatial differentiation characteristics of the levels of ecological resilience, economic resilience, and social resilience of countries along “the Belt and Road” are quite different. In countries with high levels of economic development, their comprehensive resilience is significantly higher than that of countries with low levels of economic development. There is no inevitable connection between a country’s economic growth rate and its comprehensive resilience level. At the same time, the relationship between resource richness and comprehensive resilience of countries is not apparent, but for those countries that are over-dependent on resources, the level of resilience is generally below. There is a certain degree of correspondence between urbanization rate and comprehensive resilience, that is, the comprehensive resilience will increase with the increase of urbanization rate. When the urbanization rate rises to a certain level, the level of comprehensive resilience does not change much. In this study, it provides scientific guidance for enriching regional resilience and national sustainable development theory, solving the fragile ecological environment foundation of “the Belt and Road” countries, speeding up the transformation of economic growth mode and dealing with a series of social problems.

## 1. Introduction

With the continuous advancement of the process of globalization, while the economy and society of countries around the world are becoming more and more prosperous, they are also faced with uncertainties and the interference of various crises and challenges. With the proposal of “the Belt and Road” initiative, exchanges and cooperation among countries along the route have been strengthened, and this initiative is also a meaningful way to achieve a community with a shared future for humankind. Under the guidance of this initiative, “the Belt and Road” countries have achieved rapid development and win-win cooperation. However, there are considerable differences in environmental background conditions and social and economic bases in each country. At the same time, the further development of these countries has been severely restricted by the interference of human and natural factors such as global financial crises, terrorist attacks, sandstorms, droughts and floods caused by extreme weather. The research on resilience has its unique advantages in dealing with the crises and challenges caused by uncertain factors and various human and natural factors. At the same time, this study is also a meaningful way to achieve stability, harmony, and long-term sustainable development of all countries.

Concerning the study of resilience, the ecologist Holling first put forward the concept of ecological resilience [1]. He brought this concept to the public's field of vision in the 1960s, and then the concept of resilience was applied to many fields such as economics, sociology, psychology, planning, and so on. Different disciplines have a different understanding of the connotation of resilience. The concept of resilience has roughly experienced the evolution process from the engineering resilience of a single system to the ecological resilience of a complex system and then to the social-ecological resilience [2–4]. Subsequently, the research on resilience has gradually attracted the attention of scholars from various countries. Alberti and Resilience Alliance believe that system resilience refers to the ability of a region to absorb or defend against these adverse factors and maintain the stable operation of the system when it suffers from crisis or external interference [5–7]. The research on different scales of resilience mainly includes the following, namely, community resilience [8,9], urban resilience [10–12], and regional resilience [13–15], and so on. Due to the differences in the development stage between developed and developing countries, western countries begin to pay more attention to social problems and human development, so the research on community resilience begins to increase in these countries. In developing countries, they focus more on resilience on the urban scale and regional scale, especially on the regional economic resilience. At present, there are mainly two different methods to measure resilience. The first approach is by building an indicator system. For example, in 2012, the United Nations disaster reduction Programme built a top 10 indicator system to make cities more resilient. The resilience Index has been developed by the Regional Institute of the State University of New York at Buffalo, and the University of California, Berkeley uses this index system to measure the resilience of the region, and so forth [16]. The second method is based on the equilibrium theory of cognitive thinking, by selecting a core variable to observe the changes of the core variable before and after the crisis or disturbance, in order to express the resilience level of the region under the internal and external disturbance. This method is mainly used in the measurement of economic resilience, in which the core variables include: the number of employees, GDP, the rate of change of output value [17–19], etc. With the shift of the cognition of resilience from equilibrium theory to evolution theory, the selection of core variables in measurement has changed greatly. Based on these measurement methods, scholars have measured and studied the resilience of different regions [20,21]. Research on spatial heterogeneity is also abundant [22,23]. However, their studies may be more reasonable if they had considered this situation, for example, the lack of research on a wide range of areas, especially the research on the resilience of “the Belt and Road” countries. At the same time, the current research is mainly to deal with the impact of the financial crisis, extreme weather, natural disasters and other factors on urban areas, the index is relatively single, ignoring the relationship between social, economic and ecological subsystems, and lack of research on comprehensive regional resilience [24–26].

The initiative of “the Belt and Road” has been gradually accepted by more and more countries. Up to now, it has become an international cooperation framework covering 65 countries and regions across three continents of Asia, Africa, and Europe. Although the “the Belt and Road” initiative has promoted the rapid development of countries along the route, the interference from natural and human factors such as climate change, political instability, terrorist attacks, financial crisis and unbalanced development among countries is still very significant, and there are certain differences in the crises and challenges faced by these countries. The current research on the “Belt and Road” mainly focuses on trade, transportation, etc. These studies are difficult to solve the problems faced by countries along “the Belt and Road” [27,28]. Therefore, the premise for countries to formulate development plans and select investment regions and partners according to their own actual conditions is to carry out a study on the resilience of “the Belt and Road” countries. It is also conducive to the transformation and development of the countries along the route, and is an important way to achieve sustainable development and an important guarantee for the construction of “the Belt and Road” cooperation framework. In this study, based on the summary of related concepts and connotations, combined with the specific development of “the Belt and Road” countries, the Entropy method and multi-index comprehensive evaluation method were used to measure and analyze the level of comprehensive resilience, ecological resilience, economic resilience and social resilience of “the Belt and Road” countries. In order to further explore the main influencing factors of the comprehensive resilience level, this paper uses multiple linear regression analysis to study its relationship with the degree of regional development and the level of urbanization. Then put forward corresponding countermeasures and suggestions based on the research conclusions. This study can improve the comprehensive resilience of “the Belt and Road” countries and provide decision-making for realizing mutual benefit and win-win situation of these countries and building a community with a shared future for humankind.

## 2 Study area

**Fig 1:**
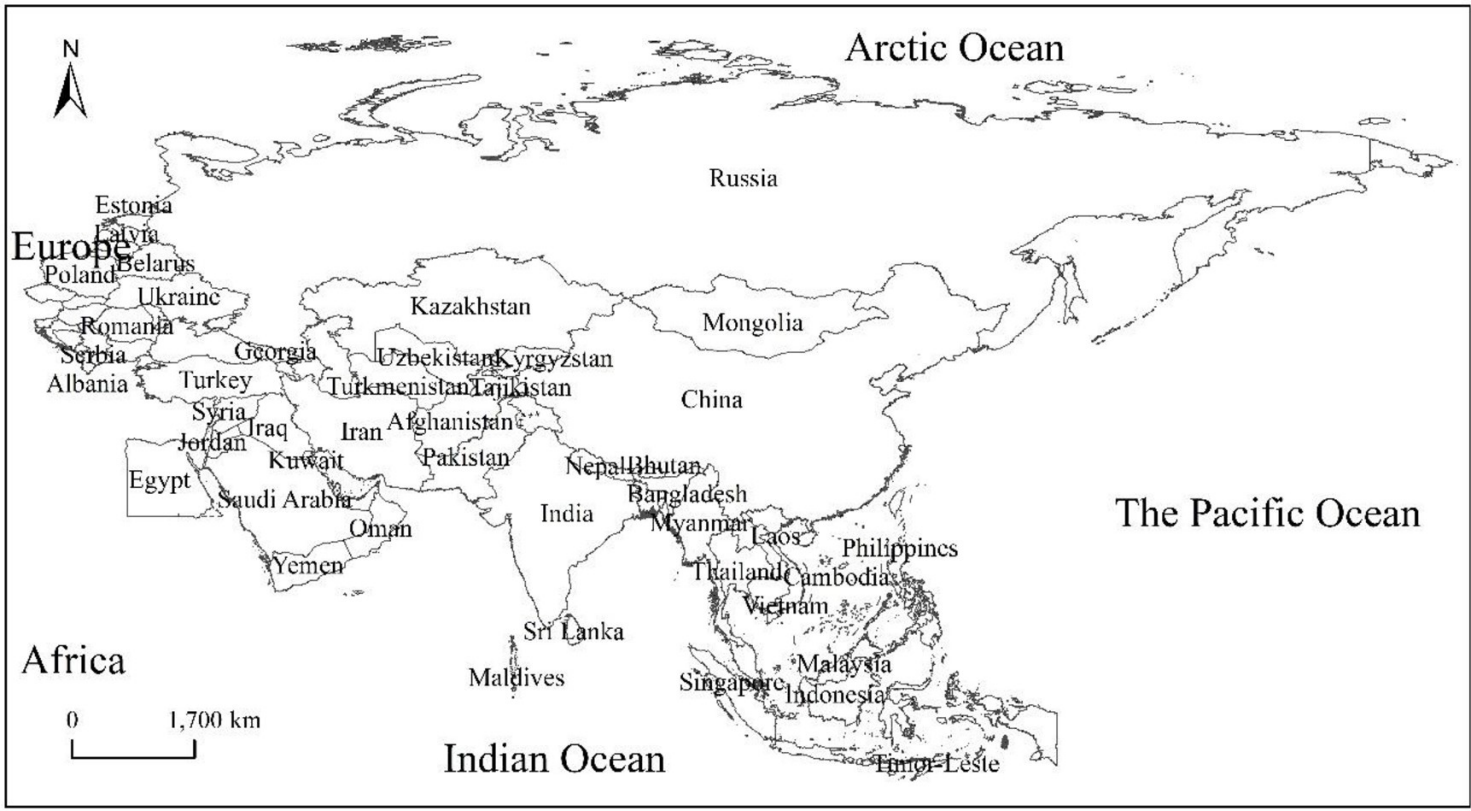
Location map of countries along “the Belt and Road”.

In this study, 65 countries along the “the Belt and Road” route are selected as the object of study (Fig 1). The area studied extends to the Arctic Ocean in the north, Africa, and inland Europe in the west, the Indian Ocean and Oceania in the south, and the Bering Strait and North America in the east. The natural and cultural environment of “the Belt and Road” countries is relatively complex and diverse. In terms of nature, these countries have diverse climate types, covering all five climatic zones, ranging from tropical to cold zones, monsoon climates to desert climates. In addition, the topography of these countries is also varied, ranging from majestic mountains to plateaus, plains, and mountains. Besides, the soils in these countries are fertile, covering more than 30 soil types [29,30]. In terms of social economy, in 2016, the total GDP of the 64 “the Belt and Road” countries except China was about the US $12 trillion, accounting for 16.0% of the global GDP; the total population was about 3.21 billion, accounting for 43.4% of the world's total population; and the total volume of foreign trade (imports + exports) was about the US $7.18856 trillion, accounting for 21.7% of the total global trade. The above data shows that although these countries have large populations, the level of economic development is relatively lagging. As the sponsor of the “the Belt and Road” initiative, China's GDP has increased from 200 billion US dollars at the beginning of reform and opening up to 11 trillion US dollars, and its share of the world has risen from 5% to 15%. Since the 2008 financial crisis, China has contributed an average of 30% to world economic growth. at the same time, China's great-leap-forward development and huge volume have injected new vitality into the countries along the route and led to the rapid development of “the Belt and Road” countries.

## 3. Materials and methodology

### 3.1 A comprehensive framework for the measurement of resilience

The concept of regional resilience is mainly based on the concept of resilience. Based on the different understandings of the concept of resilience, scholars also have different definitions of regional resilience [31–33]. A region or country is a coupling system of resources, environment, economy, and society, and the process of regional development is the process of human utilization and transformation of nature. In regional development, economic, social, resource, and environmental problems are the fundamental embodiment of restricting regional resilience. Combined with the discussion of regional resilience by relevant scholars in the past, this paper holds that: regional resilience refers to the resilience of a country or region to resist the interference of internal and external natural factors and human factors such as ecological environment, social economy and so on in the process of development. When this resilience falls below a certain critical threshold, the country or region will be in a fragile state. Regional resilience is the comprehensive embodiment of ecological resilience, social resilience, and economic resilience of a country or region.

### 3.2 Index system for comprehensive measurement of resilience

Comprehensive resilience refers to the resilience of the ecological, economic and social complex system, which mainly reflects the ability of a country or region to resist these disturbances and to maintain and restore to the normal operation of the system when it suffers from external interference. Comprehensive resilience is composed of ecological resilience, economic resilience, and social resilience. Ecological environment and economic and social development are closely related and inseparable as a whole. The ecological environment provides conditions for economic development and human survival. In order to meet the needs of economic development and human social life, human beings will continue to develop, utilize, and improve the ecological environment. The relationship between the three is interdependence and mutual promotion, but in different periods of development, there are also some contradictions and conflicts among the three. Based on the study of resilience by several scholars [34–36], 24 specific evaluation indexes corresponding to them are established from the three dimensions of ecological resilience, economic resilience, and social resilience, and the comprehensive evaluation index system of the “Belt and Road" countries resilience is constructed (Table 1).

**Table 1:** Comprehensive evaluation index system for the resilience of countries along the “Belt and Road”.

Target layer	Criterion layer	Indicator layer
Ecological Resilience	Natural resources index	Per capita arable land area (+)
		Per capita renewable freshwater resources (+)^①^
		Proportion of alternative and nuclear energy (+)
		GDP unit energy consumption (-)
	Natural environment index	Forest cover rate (+)
		Proportion of land and marine protected areas (+)
		PM_2.5_ exposure (-)^②^
		CO_2_ emissions per capita (-)
Economic Resilience	Economic development index	GDP per capita (-)
		Total reserves (-)^③^
		Employment in the service sector (+)
	Economic structure index	Proportion of value added of tertiary industry (+)
		Merchandise trade volume (+)
	Economic innovation index	R&D researchers per million people (+)
		R&D expenditure as a percentage of GDP (+)
		High-tech exports as a share of manufactured goods (+)
Social Resilience	Human development index	Life expectancy at birth (+)^④^
		College enrollment rate (+)
		Hospital beds (+)
	Infrastructure level	Internet server per million people (+)
		Railway density (+)
	Social environment index	Dependency ratio (-)^⑤^
		Unemployment rate (-)
		Gini coefficient (-)^⑥^

(+) indicates positive index, (-) indicates negative index in Table 1.

① Renewable inland freshwater resources refer to renewable resources (inland rivers and surface water generated by rainfall) in a country. The World Bank's population estimates are used to calculate renewable inland freshwater resources per capita.

② The total reserves include the currency gold held by the country, the special drawing rights (SDR), the reserves of IMF member countries held by IMF, and foreign exchange assets under the control of the monetary authorities. The value of the gold component in these reserves is based on the price of London at the end of December 31. The figures are in current dollars.

③ Life expectancy at birth refers to the number of years a newborn is likely to survive, assuming that the mortality pattern at birth remains the same throughout his or her lifetime.

④ The dependency ratio is the ratio of the dependent population (those under 15 or over 64) to the working-age population (those aged 15–64). The data is expressed as the proportion of the dependent population per 100 working-age population.

⑤ The Gini coefficient is a measure of how far an individual's or household's income distribution (and consumer spending in some cases) deviates from a perfectly even distribution in an economy. The Lorentz curve shows the relative relationship between the cumulative percentage of total income and the cumulative number of income earners, starting with the poorest individuals or households. The Gini coefficient measures the area between the Lorentz curve and the assumed absolute average, expressed as the proportion of the maximum area below that line. Therefore, a Gini coefficient of 0 indicates a complete average, and 100% indicates complete inequality.

Among them, ecological resilience is mainly reflected by the state of natural resources and the natural environment. For the per capita cultivated land area and per capita renewable inland fresh water resources, it can reflect the amount and security degree of the necessary resources for production and life. The proportion of alternative energy and nuclear energy can reflect the ability of a country or region to withstand this risk when conventional resources and energy supply are affected; GDP unit energy consumption reflects the level of resource and energy consumption of economic development. The resilience of the environment is characterized by the data of forest coverage, the proportion of wetlands and marine protected areas, PM_2.5_ exposure, and per capita carbon dioxide emissions. Among them, the forest coverage and the proportion of wetlands and marine protected areas can reflect the ability of a country or region to absorb and purify pollutants. The higher the forest coverage and the larger the proportion of wetland and marine protected areas, the stronger the area's ability to absorb and purify pollutants, and the stronger the ecological environment's resilience. PM_2.5_ exposure and per capita carbon dioxide emissions can mainly reflect the pollution of the ecological environment, when the higher the exposure of PM_2.5_ and the more carbon dioxide emissions per capita, it can show that the more serious the pollution of the ecological environment, and the less conducive to the restoration of the ecological environment.

Economic resilience is mainly reflected by three different indexes: economic development index, economic structure index, and economic innovation index. The economic development index includes per capita GDP, total reserves, and the proportion of employed people in the service sector. Among them, the per capita GDP and the proportion of the employed population in the service industry can directly show the level of economic development. Generally speaking, when the per capita GDP is higher, the proportion of the employed population in the service industry is higher, the level of economic development will be higher, and its ability to resist economic risks will be stronger. In the event of an economic crisis, the greater the total reserves, the more resilient they will be. The economic structure index includes the proportion of the added value of the tertiary industry and the volume of commodity trade, among which, the higher the proportion of the added value of the tertiary industry, the more the trade volume of goods, which indicates that the more advanced the economic structure is, the faster the economic recovery will be. The economic innovation index includes R&D researchers per million people, the proportion of scientific research expenditure in GDP, and the proportion of high-tech exports in manufactured goods. The higher the number of scientific research personnel and the proportion of R&D expenditure, the more it contributes to the application of new products and technologies. In addition, the higher the proportion of high-tech exports in manufactured products, the stronger the ability to innovate the economy, and the more able to maintain economic vitality during economic downturns.

In terms of social resilience, it includes the human development index, the level of infrastructure, and the state of the social environment. Among them, the human development index is characterized by the life expectancy at birth, the enrollment rate of colleges and universities and the number of hospital beds, in which the higher the life expectancy at birth and the more the number of hospital beds, the higher the standard of living and medical care; the higher the enrollment rate of colleges and universities, the more abundant educational resources, the more able to train high-quality talents. The higher the level of human development, the more conducive to the promotion of social resilience. In the level of infrastructure, including the number of Internet servers per million people and the density of railways, these two indicators can reflect the level of software infrastructure and hardware infrastructure of a country or region. When a country is suffering from crises and disasters, the popularity of the Internet can quickly issue early warning information and allow people to take corresponding preventive measures in advance. The improvement of transport facilities and efficient operation can get timely assistance in the event of a crisis, providing strong support for rapid recovery in crises and disasters. In the social environment, it includes the dependency ratio, unemployment rate, and Gini coefficient. Among them, the dependency ratio is the proportion of the non-working-age population to the working-age population, and the larger the dependency ratio is, the more substantial the social burden will be, which is not conducive to the recovery of the society, while the Gini coefficient represents the social income gap. This coefficient can reflect the degree of social stability. At the same time, the higher the unemployment rate, the less conducive to the stable development of society, which is not conducive to the improvement of social resilience.

### 3.3 The source of the data

In the comprehensive measure of regional resilience, the GDP used is the current price GDP of 2015. The area of cultivated land per capita comes from the (FAO, http://www.fao.org/home/en/) of the Food and Agriculture Organization of the United Nations. The data of per capita renewable inland freshwater resources, forest coverage, the proportion of wetlands and marine protected areas, and PM_2.5_ exposure come from the United Nations Environment Programme (UNEP, https://www.unenvironment.org/). The data of per capita GDP, R & D researchers per million people, the proportion of R & D expenditure in GDP, the total reserve, the proportion of employed people in the service industry and the added value of the tertiary industry all come from the Organization for Economic Cooperation and Development (OECD, https://stats.oecd.org/) and the Asian Development Bank (ADB, https://www.adb.org/). The data of merchandise trade volume, the proportion of high-tech exports to manufactured goods, the enrollment rate of colleges and universities, the Internet server per million people, the dependency ratio and the unemployment rate, come from the (WTO, https://www.wto.org/english/res_e/statis_e/statis_e.htm) of the World Trade Organization and the (ESCAP, https://www.unescap.org/) of the United Nations ESCAP. Data such as the proportion of alternative energy and nuclear energy, GDP unit energy consumption, per capita carbon dioxide emissions, life expectancy at birth, number of hospital beds, railway density, and Gini coefficient are from the World Bank (WB, https://data.worldbank.org.cn/).

### 3.4 A model method for measuring resilience

#### 3.4.1 Entropy weighting method

In order to avoid any influence on the evaluation results caused by individual subjective factors, in this paper, the entropy method commonly used in the objective weighting method is used to assign weights to the criterion layer and the index layer [37]. The specific calculation steps of the entropy method are as follows:

The first step, construct the original index data matrix. There are a total of *m* samples, and *X*_*ij*_ represents the index value of the *j-th* index of the *i-th* country.

The second step, standardization of data. The data is processed accordingly using range standardization to eliminate the impact of the original data dimension and convert it into a comparable data sequence.

Positive evaluation index, its function is:

$$Y_{ij}=(X_{ij}-X_{j\min})/(X_{j\max}-X_{j\min})$$ (1)

Negative evaluation index, its function is:

$$Y_{ij}=(X_{j\max}-X_{ij})/(X_{j\max}-X_{j\min})$$ (2)

In the formula: *X*_*ij*_ is the statistical value of the indicator; *X*_*jmax*_ and *X*_*jmin*_ are the maximum and minimum values of the same indicator, *i* is the *i*-th country, and *j* is the *j*-th indicator.

The third step, calculate the proportion *P*_*ij*_ of the index value of the *i*-th country under the *j*-th index.

$$P_{ij}=Y_{ij}/\overset{m}{\underset{i=1}{\sum }}Y_{ij}$$ (3)

The fourth step, calculate the information entropy *E*_*j*_ of the *j*-th index.

$$E_{j}=-k\overset{m}{\underset{i=1}{\sum }}P_{ij}\ln P_{ij},\;k=1/\ln m$$ (4)

The fifth step, calculate the utility value *D*_j_ of the *j*-th index.

$$D_{j}=1-E_{j}$$ (5)

The sixth step, calculate the weight of the *j*-th index.

$$W_{j}=D_{j}/\sum D_{i}$$ (6)

#### 3.4.2 Multi-index comprehensive evaluation method

First of all, the standardized value of each index in the comprehensive evaluation index system of resilience is multiplied with its weight, and any sum is made to get the ecological resilience, economic resilience and social resilience index of “the Belt and Road” countries, and then the comprehensive resilience index is obtained by weighted summation. The specific calculation formula is as follows:

$$CR_{c}=\sum_{i=1}^{n}r_{i}w_{i}$$ (7)

$$CR=\overset{m}{\underset{j=1}{\sum }}{\left(CR_{c}\right)}_{j}w_{j}$$ (8)

In the formula: *CRc* represents the index of ecological resilience, economic resilience, and social resilience, *CR* represents the index of comprehensive resilience, *w*_*i*_ represents the weight of the index, *n* represents the number of indicators contained in the criterion layer, *r*_*i*_ represents the quantitative index value of the index, *m* represents the number of criterion layers, and *w*_*j*_ represents the weight of the elements of the criterion layer.

#### 3.4.3 Grading standard of resilience

At present, there are few studies on resilience. There is a particular relationship between resilience and vulnerability, and the two usually appear as opposite concepts. The increase in resilience will inevitably lead to a reduction in vulnerability [38,39]. Furthermore, the concept of resilience is similar to the concepts of elasticity and resilience. By referring to the classification criteria of vulnerability, elasticity, and toughness [10,40,41], the resilience index is divided into five levels, which are low resilience, relative low resilience, moderate resilience, relative high resilience and high resilience (Table 2).

**Table 2:** Classification criteria for resilience.

Classification low resilience relative low resilience moderate resilience relative high resilience high resilience
Index R 0≤R<0.1 0.1≤R<0.3 0.3≤R<0.5 0.5≤R<0.7 0.7≤R<1

## 4. Results

### 4.1 Calculation and analysis of index weight coefficient

First of all, through the use of entropy weighting method to calculate the comprehensive resilience target layer of “the Belt and Road” countries and the weight of specific index factors (Table 3). In the target layer of comprehensive resilience of “the Belt and Road” countries, the weight coefficient of economic resilience is the largest (0.4838), the second is ecological resilience (0.3280), and the smallest is social resilience (0.1883). In the index layer, the weight coefficient is also significantly different.

**Table 3:** Weight of comprehensive resilience target layer and index layer along “the Belt and Road”.

Target layer	Weights	Criterion layer	Indicator layer	Weights
Ecological Resilience	0.3280	Natural resources index	Per capita arable land area (+)	0.0016
			Per capita renewable freshwater resources (+)	0.0504
			Proportion of alternative and nuclear energy (+)	0.1018
			GDP unit energy consumption (-)	0.0835
		Natural environment index	Forest cover rate (+)	0.0387
			Proportion of land and marine protected areas (+)	0.0040
			PM_2.5_ exposure (-)	0.0451
			CO_2_ emissions per capita (-)	0.0029
Economic Resilience	0.4838	Economic development index	GDP per capita (+)	0.0458
			Total reserves (+)	0.1975
			Employment in the service sector (+)	0.0084
		Economic structure index	Proportion of value added of tertiary industry (+)	0.0107
			Merchandise trade volume (+)	0.0278
		Economic innovation index	R&D researchers per million people (+)	0.0691
			R&D expenditure as a percentage of GDP (+)	0.0704
			High-tech exports as a share of manufactured goods (+)	0.0540
Social Resilience	0.1883	Human development index	Life expectancy at birth (+)	0.0165
			College enrollment rate (+)	0.0238
			Hospital beds (+)	0.0154
		Infrastructure level	Internet server per million people (+)	0.1061
			Railway density (+)	0.0055
		Social environment index	Dependency ratio (-)	0.0057
			Unemployment rate (-)	0.0053
			Gini coefficient (-)	0.0010

### 4.2 Overall evaluation of resilience of “the Belt and Road” countries

The comprehensive resilience, ecological resilience, economic resilience, and social resilience of “the Belt and Road” countries were measured by using a multi-index comprehensive evaluation method. In terms of the comprehensive resilience level of “the Belt and Road” countries, Singapore has the highest level of comprehensive resilience, reaching 0.414, followed by Slovenia (0.406), China (0.380), the Czech Republic (0.342), Estonia (0.325), Israel (0.320) and Slovakia (0.308). The rest of the country is at a relatively low level of resilience and a low level of resilience. A total of 7 countries are at a moderate level of resilience, accounting for 10.8% of the total number of “the Belt and Road” countries, while countries with relatively low and low levels of resilience account for 89.2%.

From the level of ecological resilience of “the Belt and Road” countries, only Bhutan and Slovenia reached a higher level, and their resilience indexes were 0.569 and 0.502, respectively. Secondly, countries such as Slovakia, Bulgaria, Tajikistan, Russia, and Albania reached a moderate resilience level, with resilience indices of 0.391, 0.373, 0.366, 0.333, and 0.311, respectively. The resilience of the rest of the country is at a relatively low level and at a low level. In general, the level of ecological resilience of “the Belt and Road” countries is generally on the low side.

From the level of economic resilience of “the Belt and Road” countries, China has the highest economic resilience, with a value of 0.593, which is at a relatively high level, followed by Singapore and Israel, whose economic resilience indices are 0.436 and 0.391 respectively, both at a moderate level. The economic resilience index of other countries is less than 0.3. In terms of quantity, countries with a relatively low level of economic resilience and a low level of resilience account for 95.4% of the total in the “the Belt and Road” countries.

From the level of social resilience of “the Belt and Road” countries, the countries with high resilience are Singapore and Estonia, and their resilience indexes are 0.970 and 0.803, respectively. Slovenia, Czech Republic, Israel, and Latvia are countries with high resilience levels, with resilience indices of 0.689, 0.654, 0.531, and 0.500, respectively. A total of 17 countries are at moderate resilience levels, which will be higher than other levels, such as Lithuania, Poland, Slovakia, and Hungary. In terms of quantity, 69.2% of the countries with “the Belt and Road” countries have a relatively low level of social resilience and a low level of resilience.

### 4.3 Spatial heterogeneity characteristics of the resilience of “the Belt and Road” countries

**Fig 2:**
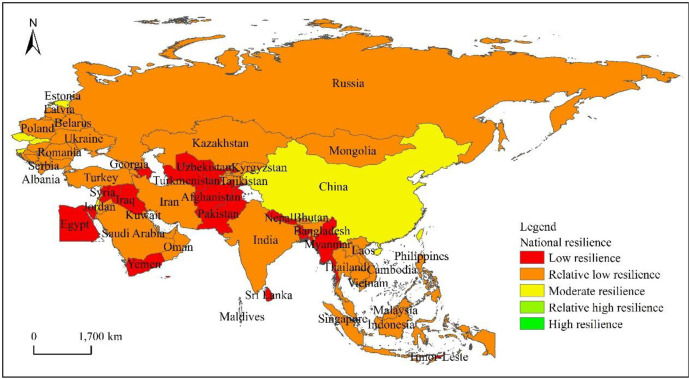
Horizontal spatial differentiation of the comprehensive resilience of countries along “the Belt and Road”.

From the perspective of the spatial heterogeneity of the “the Belt and Road” countries' comprehensive resilience levels (Fig 2), it shows a spatial distribution characteristic of “large dispersion and small agglomeration”. Countries with moderate levels of resilience are mainly distributed in East and Central and Eastern Europe, in addition to Singapore in Southeast Asia. These countries have strong comprehensive strength, and their comprehensive resilience is higher than other countries; Countries with relatively low resilience levels are widely distributed, basically in every region, while for countries with low resilience levels, they are mainly concentrated in Central Asia. South Asia, except India, and countries such as Yemen and Egypt in West Asia; there are no countries with relatively high levels of resilience and high resilience at present.

**Fig 3:**
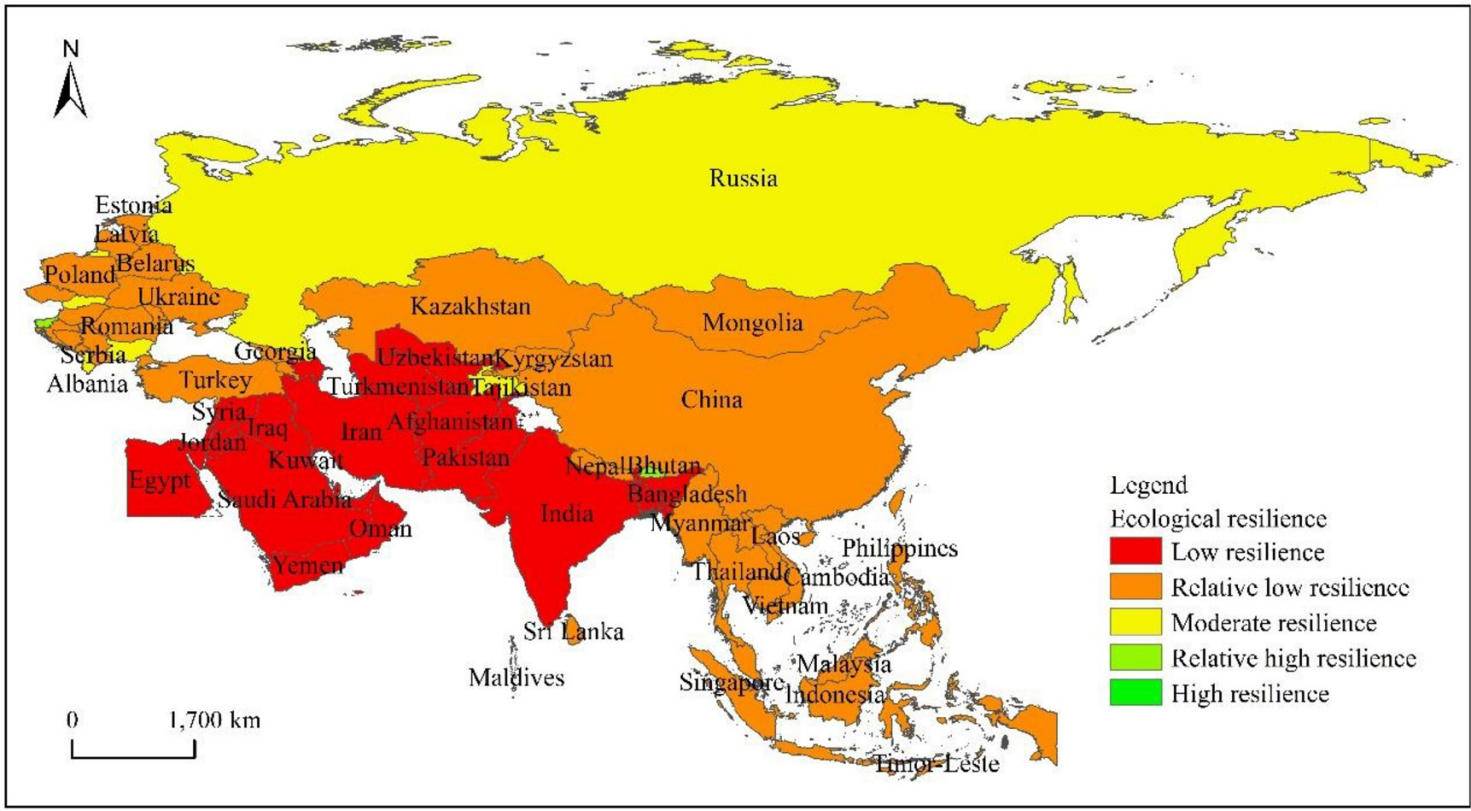
Spatial differentiation of ecological resilience in countries along “the Belt and Road”.

From the spatial heterogeneity of the ecological resilience level of “the Belt and Road” countries (Fig 3), they show the spatial distribution characteristics of “centralized connection” as a whole. Slovenia in Central and Eastern Europe and Bhutan in South Asia are countries with a high level of resilience, which shows that the ecological environment of these countries is better. The countries with moderate resilience are mainly distributed in the CIS countries led by Russia and some countries in Central and Eastern Europe. The countries with a low level of resilience are mainly distributed in Central and Eastern Europe, East Asia, and Southeast Asia, as well as Turkey in West Asia and Kazakhstan in Central Asia. Countries with a low level of resilience are mainly distributed in South Asia, Central Asia, and West Asia, indicating that their eco-environmental conditions are relatively poor.

**Fig 4:**
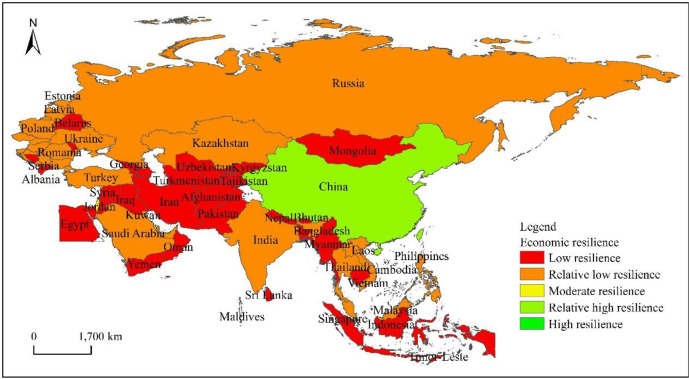
The spatial differentiation of the economic resilience of the countries along “the Belt and Road”.

According to the spatial heterogeneity (Fig 4) of the economic resilience level of “the Belt and Road” countries, the overall situation shows the characteristics of “agglomeration and dispersion cross-distribution”. Among them, among the countries in East Asia, China has the highest economic resilience at a high level, while Singapore in Southeast Asia and Israel in West Asia have moderate economic resilience. The largest number of countries are at a relatively low level of resilience, which is distributed in Central and Eastern Europe, CIS countries, Turkey, and Saudi Arabia in West Asia, Kazakhstan in Central Asia, India in South Asia, and countries in the northern part of Southeast Asia. Countries with a low level of resilience are concentrated in the south of Central and West Asia and Southeast Asia.

**Fig 5:**
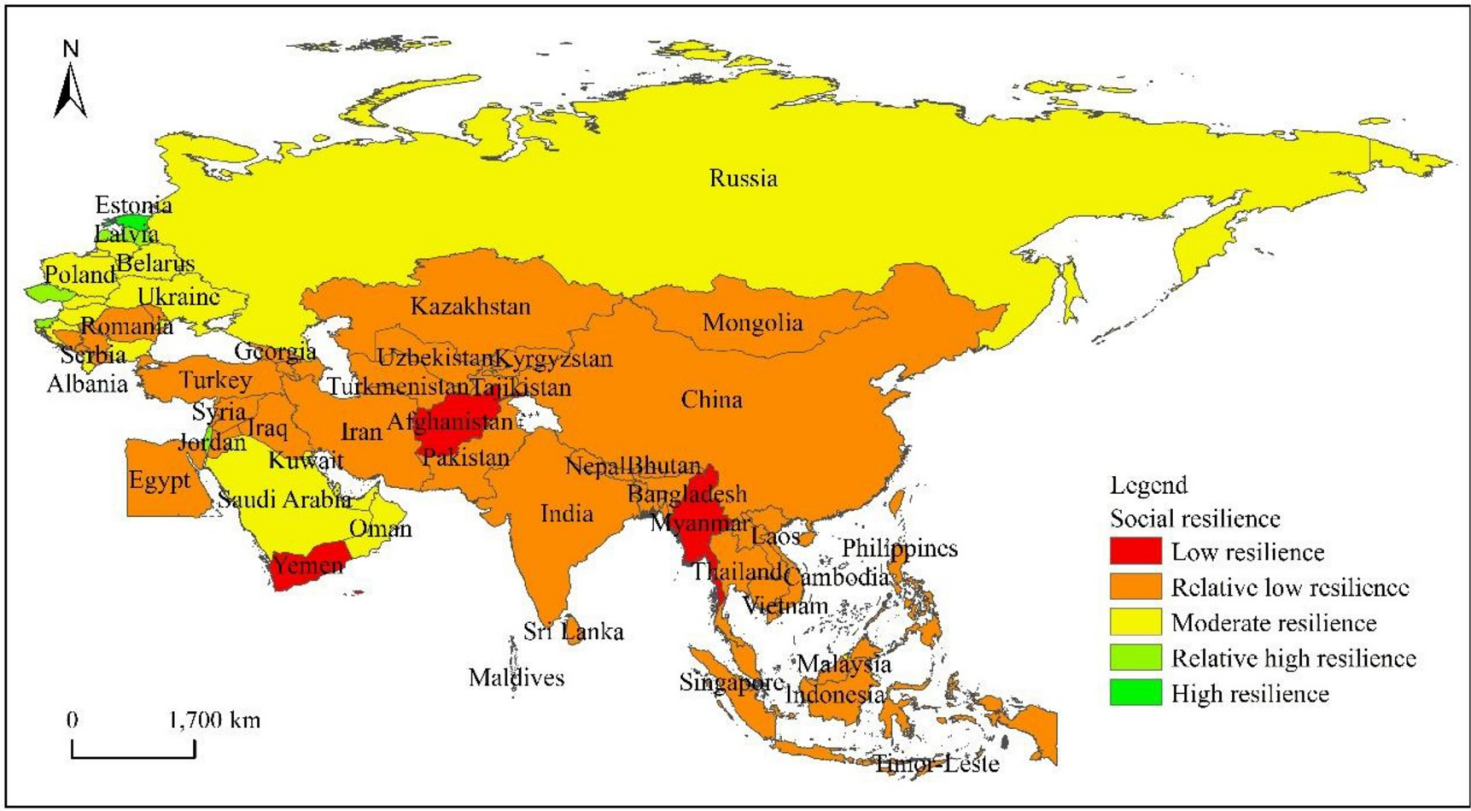
Spatial differentiation of social resilience in countries along “the Belt and Road”.

According to the spatial heterogeneity (Fig 5) of the social resilience level of “the Belt and Road” countries, it shows a significant “agglomeration” distribution characteristic as a whole. The countries with relatively high and high levels of social resilience are mainly distributed in Central and Eastern Europe, which shows that the social environment and the level of human development in this region are relatively high. Most of the countries in Central and Eastern Europe and the CIS are at moderate resilience levels; most countries in East Asia, Central Asia, West Asia, South Asia, and South-East Asia are at relative low resilience levels; while countries such as Afghanistan, Myanmar, and Yemen are at low resilience levels, which indicates that the social and environmental security systems and infrastructure levels in these countries are not yet perfect.

### 4.4 The relationship between the level of resilience and the rate of economic growth

**Fig 6:**
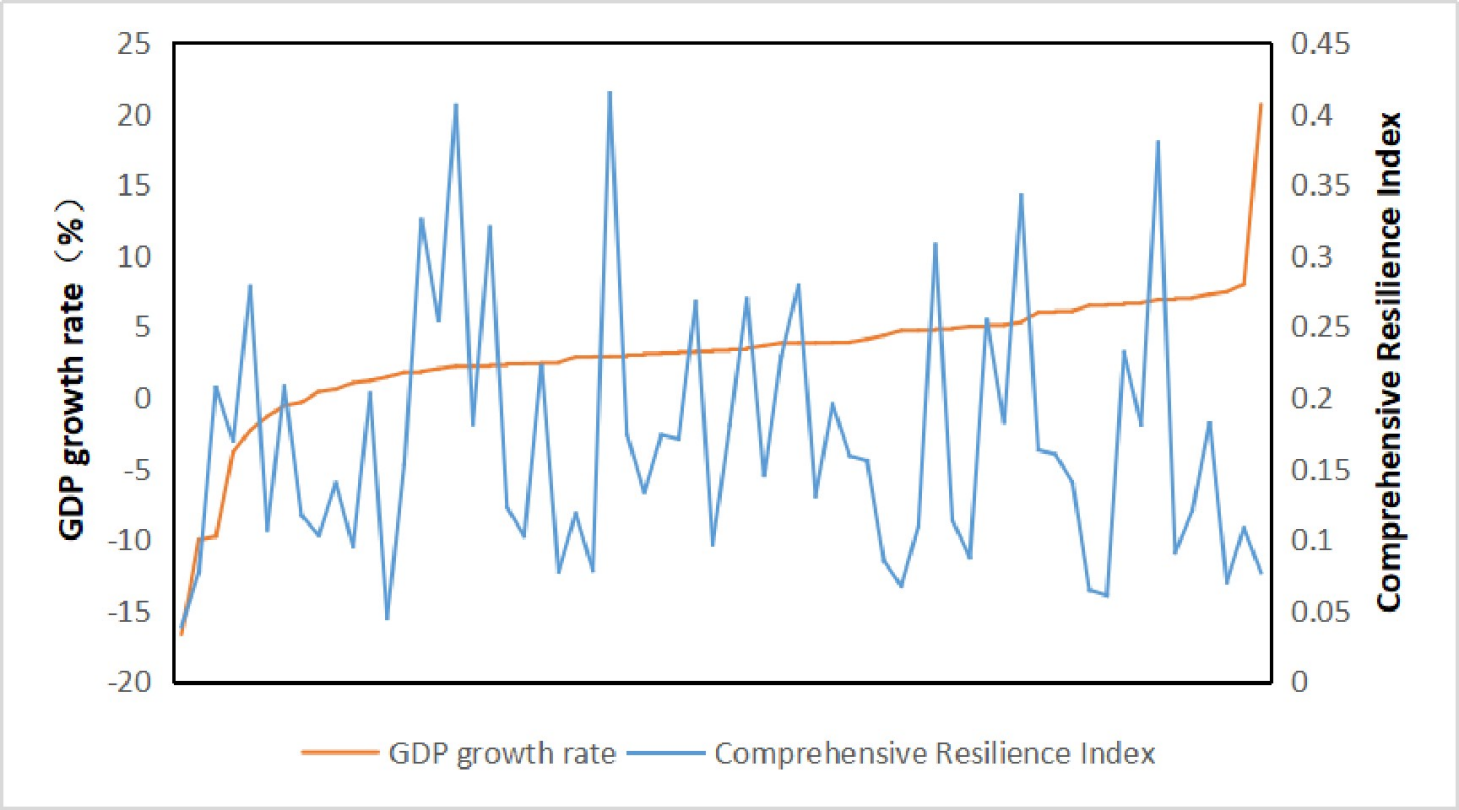
Relationship between comprehensive resilience and economic growth.

The growth rate of GDP reflects the level of economic growth so as to analyze the relationship between economic growth and resilience. In 2015, the country with the slowest GDP growth rate was Yemen, followed by Syria and Ukraine. Their GDP growth rates were -16.68%, -10.00%, and -9.77%, and their corresponding comprehensive resilience indexes were 0.0388., 0.0769, 0.2072; The country with the fastest economic growth in East Timor, followed by India and Uzbekistan, with GDP growth rates of 20.63%, 8.00% and 7.45% respectively, with corresponding comprehensive resilience indices of 0.0768, 0.1083 and 0.0701 respectively. Although their GDP growth rates vary widely among the six countries, there is little difference in the overall resilience index. Thus, it can be seen that economic growth does not reflect the level of comprehensive resilience (Fig 6). Besides, it can also be seen from Fig 6 that with the continuous rise of economic growth rate, the comprehensive resilience index shows irregular changes, but does not decrease with the increase of GDP growth rate, which shows that there is no inevitable relationship between the two. The speed of economic growth can not reflect the level of comprehensive resilience.

### 4.5 The relationship between the level of resilience and the rate of urbanization

**Fig 7:**
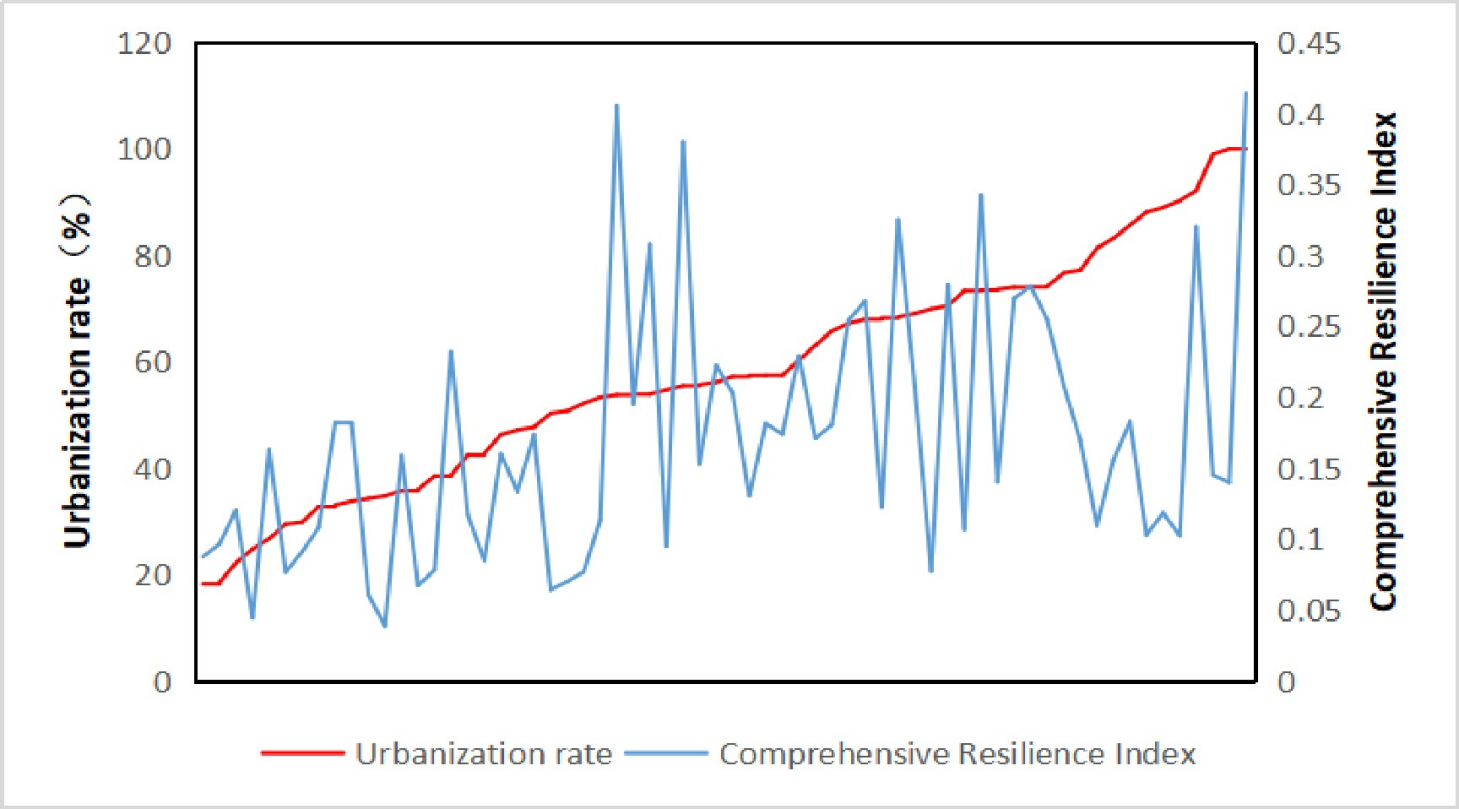
Relationship between comprehensive resilience and urbanization rate.

The urbanization rate can reflect the urbanization process of a country. The relationship between the urbanization process and comprehensive resilience can be analyzed. From the perspective of individual countries (Fig 7), there is no clear corresponding relationship between comprehensive resilience and the process of urbanization, but with the increase of urbanization rate, the comprehensive resilience index will show a trend of increasing at first and then changing little. That is to say, from a single city, for countries with a high urbanization rate, the level of comprehensive resilience is not necessarily high, and countries with not very high urbanization rates can also be suitable for economic development and human life. However, on the whole, with the continuous improvement of the level of urbanization, in the initial stage, the level of comprehensive resilience will show a significant upward trend, which shows that urbanization promotes economic development and the improvement of people's living standards. However, when urbanization enters a mature stage, with the frequent occurrence of all kinds of urban diseases, it will be challenging to improve the level of comprehensive resilience.

### 4.6 Analysis of influencing factors and mechanism

With the continuous promotion of the process of globalization, the population and industries of “the Belt and Road” countries continue to gather, which makes these countries develop rapidly in economy and society, while at the same time these countries are facing problems such as resource depletion and environmental pollution. Under the condition of resource shortage and environmental constraints, in order to further explore the main factors affecting the level of comprehensive resilience of “the Belt and Road” countries. By comprehensively considering the constituent elements of the target layer in the comprehensive resilience evaluation index system of “the Belt and Road” countries, we can analyze the eight factors that affect the level of comprehensive resilience from the aspects of natural resources, natural environment, economic development level, economic structure index, economic innovation index, human development index, infrastructure level, and social environment condition. Through the regression analysis by using multiple linear regression analysis, it can be found that the status of natural resources, the level of economic development, the ability of economic innovation and the level of infrastructure have the most apparent impact on regional resilience.

One of the essential conditions in economic development is natural resources, and because most of the industrial systems of “the Belt and Road” countries are not perfect, they need to rely on the advantages of natural resources to develop their economy. With the reduction of resources and the impact of external economic crisis, countries rich in resources have strong resilience in dealing with internal and external interference. In resilience, the most critical factor is the level of economic development, which will affect not only economic resilience, but also social resilience through education, social welfare, and social security. At the same time, through financial support to improve the environment and purchase resources and other impacts on the size of ecological resilience, when the higher the level of economic development, its diversified economic advantages will show strong resilience in response to internal and external interference. Besides, another important factor affecting resilience is the ability of economic innovation, and under the conditions of shortage of resources and environmental constraints, traditional industries with high energy consumption and high pollution can no longer adapt to the trend of globalization. At the same time, the countries with more robust economic innovation ability are more able to achieve economic transformation and development, while for countries with poor economic innovation ability, the path of transformation and development will be lack of a clear direction, which will lead to the impact. It is more difficult to recover from the crisis. For a country, the vital guarantee of its economic and social development is the perfection of infrastructure. For example, the developed network communication and its extensive coverage can provide early warning of disasters or crises, and play a decisive role in prevention in advance. Developed transport infrastructure can play a vital role in post-crisis reconstruction. In terms of the speed of recovery, countries with more developed infrastructure have apparent advantages over countries with poor infrastructure, which is one of the essential factors affecting resilience.

As for resilience, there are many influencing factors. Natural resources and environmental conditions are the carriers of economic development, and economic and social development, in turn, will have a positive impact on natural resources and the environment. The ultimate goal of economic development is to achieve social progress and people's all-round development. These factors influence each other and determine the strength of resilience. Due to the high level of economic development in countries such as Singapore, China, the Czech Republic, and Israel, these countries have more developed service industries, higher economic innovation capabilities, and sound infrastructure. Although the resilience level of “the Belt and Road” countries is generally low, it has certain advantages in these countries, so the resilience level of these countries is higher than that of other countries.

## 5 Conclusions and discussion

### 5.1 Conclusion

Through the research on the resilience of “the Belt and Road” countries, it can help to enhance the ability of countries along the route to cope with uncertainties and various crises and challenges, which is of great significance to the realization of sustainable development. In this study, the entropy method and multi-index comprehensive evaluation method are used to measure it, and its spatial heterogeneity characteristics and influencing factors are analyzed. The main conclusions are as follows:

(1) Among the resilience of “the Belt and Road” countries, the weight coefficient of economic resilience is the largest, which is 0.484, followed by that of ecological resilience, which is 0.328, and that of social resilience is the smallest, which is 0.188. In the index layer, the weight coefficient is also significantly different.

(2) The levels of comprehensive resilience, ecological resilience, economic resilience, and social resilience of “the Belt and Road” countries are generally low; furthermore, there are some differences in spatial distribution characteristics. Among them, China has a large economy, a relatively perfect industrial system, and a high level of economic resilience. In Central and Eastern Europe and other countries, their social welfare and security system is relatively perfect, and the level of social resilience is high. Russia is characterized by its vast territory and abundant natural resources, so its level of ecological resilience is high. However, if a country is overly dependent on resources, its resilience level is often low, such as some resource-rich countries in West Asia, Central Asia, and South Asia. This is similar to Fang Chuanglin's view on urban vulnerability research [40].

(3) The status of natural resources, the level of economic development, the ability of economic innovation, and the level of infrastructure are the main factors that affect resilience. The influencing factors of resilience are various, natural resources and environmental conditions are the carriers of economic development, and economic and social development, in turn, has a certain impact on natural resources and environment. The ultimate goal of economic development is to achieve social progress and people's all-round development. These factors influence each other and work together to determine the level of comprehensive resilience. This is similar to Li Bo's views on the study of urban resilience [36].

(4) From the relationship between economic growth rate, urbanization level, and comprehensive resilience, we can see that the speed of economic growth can not reflect the level of comprehensive resilience, that is, the comprehensive resilience level of countries with rapid economic growth is not necessarily high. From the point of view of the relationship between the level of comprehensive resilience and the process of urbanization, with the urbanization rate increases, the comprehensive resilience index increases first and then stabilizes.

### 5.2 Discussion

Although the resilience level of “the Belt and Road” countries has been studied in this paper, there are still some shortcomings. First of all, because it is difficult to obtain data, in this study, only the resilience level of “the Belt and Road” countries in a single year is measured, so the next step is to analyze the time evolution by combining the data of long time series. Secondly, the meticulous degree of the research needs to be more meticulous, and the small-scale resilience level can be diagnosed with the help of big data and other technical means, in order to provide a more accurate reference for decision-makers. Finally, it is necessary to strengthen the research on the coupling among ecological resilience, economic resilience, and social resilience, which is of great significance for the promotion of comprehensive regional resilience and the realization of sustainable development.

## Supporting information

S1 File (XLS)
